# Evaluating AI chatbots in penis enhancement information: a comparative analysis of readability, reliability and quality

**DOI:** 10.1038/s41443-025-01098-3

**Published:** 2025-06-03

**Authors:** Mehmet Vehbi Kayra, Hakan Anil, Ilturk Ozdogan, Suhail Mohamed Amin Baradia, Serdar Toksoz

**Affiliations:** 1 Department of Urology, Baskent University Adana Dr. Turgut Noyan Application and Research Center, Adana, Turkey; 2 Department of Urology, Adana City Hospital, Adana, Turkey; 3 Department of Urology, Sincan Training and Research Hospital, Ankara, Turkey; 4 Department of Urology, Baskent University Faculty of Medicine, Ankara, Turkey

**Keywords:** Risk factors, Sexual dysfunction

## Abstract

This study aims to evaluate and compare the performance of artificial intelligence chatbots by assessing the reliability and quality of the information they provide regarding penis enhancement (PE). Search trends for keywords related to PE were determined using Google Trends (https://trends.google.com) and Semrush (https://www.semrush.com). Data covering a ten-year period was analyzed, taking into account regional trends and changes in search volume. Based on these trends, 25 questions were selected and categorized into three groups: general information (GI), surgical treatment (ST) and myths/misconceptions (MM). These questions were posed to three advanced chatbots: ChatGPT-4, Gemini Pro and Llama 3.1. Responses from each model were analyzed for readability using the Flesch-Kincaid Grade Level (FKGL) and Flesch Reading Ease Score (FRES), while the quality of the responses was evaluated using the Ensuring Quality Information for Patients (EQIP) tool and the Modified DISCERN Score. All chatbot responses exhibited difficulty in readability and understanding according to FKGL and FRES, with no statistically significant differences among them (FKGL: p = 0.167; FRES: p = 0.366). Llama achieved the highest median Modified DISCERN score (4 [IQR:1]), significantly outperforming ChatGPT (3 [IQR:0]) and Gemini (3 [IQR:2]) (p < 0.001). Pairwise comparisons showed no significant difference between ChatGPT and Gemini (p = 0.070), but Llama was superior to both (p < 0.001). In EQIP scores, Llama also scored highest (73.8 ± 2.2), significantly surpassing ChatGPT (68.7 ± 2.1) and Gemini (54.2 ± 1.3) (p < 0.001). Across categories, Llama consistently achieved higher EQIP scores (GI:71.1 ± 1.6; ST: 73.6 ± 4.1; MM: 76.3 ± 2.1) and Modified DISCERN scores (GI:4 [IQR:0]; ST:4 [IQR:1]; MM:3 [IQR:1]) compared to ChatGPT (EQIP: GI:68.4 ± 1.1; ST: 65.7 ± 2.2; MM:71.1 ± 1.7; Modified DISCERN: GI:3 [IQR:1]; ST:3 [IQR:1]; MM:3 [IQR:0]) and Gemini (EQIP: GI:55.2 ± 1.4; ST:55.2 ± 1.6; MM:2.6 ± 2.5; Modified DISCERN: GI:1 [IQR:2]; ST:1 [IQR:2]; MM:3 [IQR:0]) (p < 0.001). This study highlights Llama’s superior reliability in providing PE-related health information, though all chatbots struggled with readability.

## Introduction

Penis enhancement has long been a topic of interest across various cultures, driven by both functional and aesthetic motivations [1–5]. A large-scale study found that 45% of men desire a larger penile length, highlighting a widespread interest in size enhancement [6]. While some seek enhancement for congenital or functional reasons, others pursue it for cosmetic purposes, often despite having anatomically normal sizes, a phenomenon linked to penis dysmorphophobia [7]. Despite the popularity of this topic, many commercially promoted treatments lack proven medical efficacy, underscoring the growing need for reliable information on the safety and effectiveness of these procedures [3, 8].

One of the innovations brought about by the digital age is artificial intelligence (AI) powered chatbots, which have become a vital resource for individuals seeking information on health-related topics [9]. These AI-based systems provide users with anonymous and quick guidance, offering a practical solution, especially for those hesitant to approach healthcare professionals [10]. Due to their accessibility and ease of use, AI chatbots are increasingly being preferred by individuals seeking guidance [10]. However, the accuracy, consistency and quality of the information provided by these chatbots is crucial to ensure that individuals are properly informed [11].

While numerous studies have assessed AI chatbot responses on andrological issues, no research has specifically focused on the accuracy and quality of responses related to penis enhancement [12–14]. On these platforms, it is essential to differentiate evidence-based treatment methods from myths and to provide accurate information about potential complications [11]. Several studies have evaluated the performance of various chatbots in providing medical information, revealing inconsistencies and limitations, especially in specialized areas such as urology and andrology [12, 13, 15–17]. This study aims to evaluate the accuracy, consistency and quality of information provided by different chatbots on the topic of penis enhancement. Additionally, it seeks to offer a detailed analysis by comparing the performance of various AI chatbots, contributing to a better understanding of the information accessed by individuals seeking guidance in this topic.

## Materials and methods

This study aims to comparatively evaluate the quality and readability of answers to frequently asked questions about “penis enhancement” in the field of urology, obtained from three different large language models. The methodological process consists of a multi-stage design, detailed as follows. This study uses publicly available online data and does not involve human participants or clinical information, so ethical review board approval was not required.

The term penile enhancement was used as an umbrella term to encompass penile girth enlargement, penile length enlargement and augmentation procedures. In the first stage, online search trends for “penis enhancement” and related keywords were identified using Google Trends (Google LLC, USA, https://trends.google.com) and Semrush (Semrush Inc., USA, https://www.semrush.com). Data covering a ten-year period was analyzed to determine search volumes and regional trends. Additional comparisons were made with data from Ahrefs (Ahrefs Pte. Ltd., Singapore, https://ahrefs.com), Moz Keyword Explorer (Moz, Inc., USA, https://moz.com/explorer), and Google Keyword Planner (Google LLC, USA, https://ads.google.com/intl/en_uk/home/tools/keyword-planner/). Keywords were used as the basis for question formulation by urology experts, who ensured that the questions were clinically relevant, clear, and representative of common patient concerns. Specific and non-informative topics, such as doctor preferences and costs, were excluded from the study. User inquiries focused on three primary subgroups: 1- General information (treatment indications, treatment types), 2-Surgical treatment (risks, complications and effectiveness), 3- Myths/misconceptions (information without medical basis). The questions were selected based on the most frequently searched topics and user inquiries. A total of 25 questions were curated for each subgroup, forming the core question set (Table 1).

**Table 1:** Comprehensive List of Questions on Penis Enhancement by Category.

**General Information**
1. Who is eligible for penile enlargement treatment?
2. What is the minimum ideal length of the male penis?
3. What methods are available to increase penile girth or length?
4. Which method is more effective for increasing penile length?
5. What is the most effective method for increasing penile girth?
6. Is the effect of penile enlargement with fillers temporary?
7. What are the risks of penile enlargement with fillers?
8. Which filler material is most suitable for penile enlargement?
9. Do massage techniques or vacuum pumps provide permanent penile lengthening?
**Surgical Treatment**
10. What is the most effective penile enlargement surgery?
11. Which penile lengthening surgery has the lowest risk?
12. Do penile enlargement surgeries provide permanent results?
13. What is the recovery process after penile surgery, and when can sexual activity be resumed?
14. Are penile implants or prosthetics a solution for enlargement?
15. What are the most common complications of penile enlargement surgery?
16. Can penile lengthening surgery affect erection quality, sensation or penile angle?
17. How can the risks of penile enlargement surgery be minimized?
**Myths/Misconceptions**
18. Do herbal supplements have any effect on penile enlargement?
19. Can natural oils or herbal creams increase penile size over time?
20. Can any exercise provide permanent penile growth?
21. Is it true that consuming certain foods can increase penile size?
22. Are pills or creams marketed for penile enlargement reliable?
23. Is heat application effective for penile enlargement?
24. Does dietary change affect penile size?
25. Which medications can increase penile length?

In the second stage, these questions were posed to three chatbots (ChatGPT-4, Gemini Pro and Llama 3.1 Large). ChatGPT-4 and Gemini Pro were chosen for their widespread use in research, and Llama for its research-oriented infrastructure developed by Meta [18]. To eliminate potential biases before the process, new user accounts were created on these bots using new email addresses. Responses for the 25 questions per model were requested in “raw text” format without any additional guidance. All answers were documented in a table on Microsoft Word or Google Docs in the format “Question – Model – Answer Text” compiling a total of 75 responses in total across the three models. To ensure data accuracy, a second researcher cross-verified the transcribed texts.

Quantitative and qualitative assessments were conducted on the collected responses. Metrics such as word count (WC), sentence count (SC) and syllable count (SYC) were automatically calculated. Readability was assessed using the Flesch Reading Ease (FRES) and Flesch-Kincaid Grade Level (FKGL) metrics [19]. The FRES score, ranging from 0 to 100, indicates how easy a text is to read, with scores between 80 and 100 reflecting easy readability and scores from 0 to 30 indicating more challenging texts. The FKGL score estimates the educational level required to understand the text, where lower scores (0–6) align with elementary school levels, and scores above 12 correspond to university-level difficulty [19]. Both metrics are derived from specific formulas:

FRES formula = 206.835 − (1.015 × WC/SS) − (84.6 × WCS/YC).

FKGL formula = (0.39 × SS/WC) + (11.8 × WC/SYC) −15.59.

Both scores were calculated using Python’s textstat or R-based libraries to ensure consistent analysis.

The Ensuring Quality Information for Patients (EQIP) tool was used to evaluate the accuracy, completeness and timeliness of urological information in the responses. The EQIP tool consists of 20 questions with response options “yes,” “partly yes,” “no,” and “does not apply” [20]. Scores were calculated using the formula:

$$\frac{{EQIP\; Score}=(({yes}\times 1)+({partly}\times 0.5)+({no}\times 0))\,}{\,(20-{does\; not\; apply})\times 100}$$

The results were classified into four categories:

- 0–25%: Severe quality problems
- 26–50%: Serious quality issues
- 51–75%: Good quality with minor issues
- 76–100%: Well written

Lastly, the Modified DISCERN Score was utilized to measure the quality of health-related information [21, 22]. Health-related responses are rated on a scale from 1 to 5. A score of 0–1 indicates poor quality with misleading information. A score of 2 represents poor quality with incomplete information. A score of 3 reflects fair quality, where the information is basic and mostly accurate. A score of 4 shows good quality, with mostly accurate and reliable information. A score of 5 signifies excellent quality, providing fully accurate and comprehensive information. The final scores were determined through a double-blind review by authors HK and MK, with a third evaluator, ST, resolving discrepancies.

### Statistical analysis

Descriptive statistics were presented as median (IQR) for non-parametric data and mean ± standard deviation for parametric data. Comparisons among the three groups were conducted using one-way ANOVA for normally distributed variables and the Kruskal-Wallis test for variables that did not meet the normality assumption. Post hoc analyses were performed using the Bonferroni correction following one-way ANOVA and pairwise comparisons following the Kruskal-Wallis test in cases where statistical significance was detected. All analyses were conducted using SPSS version 27 (IBM Corp., Armonk, NY, USA). A p-value < 0.05 was considered indicative of statistical significance.

## Results

In the first stage, the responses provided by the three chatbot models—ChatGPT, Gemini, and Llama—were evaluated in terms of word count, sentence count and readability using the FRES and FKGL scores. The average sentence count per response was 9.7 ± 5.5 for ChatGPT, 9.7 ± 4.6 for Gemini, and 10.8 ± 4.7 for Llama (p = 0.416). The average word count per response was determined to be 116.2 ± 39.4 for ChatGPT, 137.4 ± 65.6 for Gemini and 190.5 ± 71.2 for Llama (p < 0.001). Post hoc analysis revealed no statistically significant difference in word count between ChatGPT and Gemini (p = 0.658). However, Llama exhibited a statistically significant higher word count per sentence compared to ChatGPT and Gemini (p < 0.001 for both). Readability was assessed using FKGL and FRES scores, but neither measure showed statistically significant differences between the groups (p = 0.167 for FKGL; p = 0.366 for FRES).

**Fig. 1:**
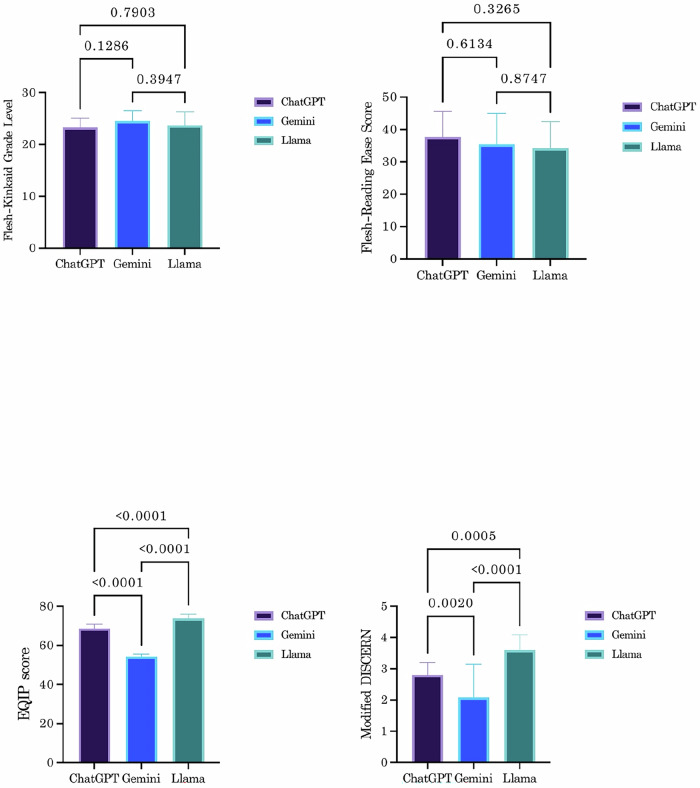
Chatbot evaluation: Pairwise comparison of readability and quality scores.

In the second stage of analysis, the quality and reliability of the responses provided by the chatbots were evaluated using the Modified DISCERN and EQIP scoring methods. The median (IQR) values for Modified DISCERN scores were 3 (0) for ChatGPT, 3 (2) for Gemini, and 4 (1) for Llama (p < 0.001). Pairwise comparisons revealed no statistically significant difference between ChatGPT and Gemini (p = 0.070). However, Llama outperformed both ChatGPT and Gemini in Modified DISCERN scores, with statistically significant differences (p < 0.001 for both). In EQIP scores, Llama also achieved the highest average score (73.8 ± 2.2), significantly outperforming ChatGPT (68.7 ± 2.1, p < 0.001) and Gemini (54.2 ± 1.3, p < 0.001). The comparative analyses of scoring methods across groups and between pairs of groups are summarized in Table 2 and Fig. 1.

**Table 2:** Comparative group analysis of ChatGPT, Gemini and Llama chatbots in terms of information quality and readability metrics.

Variables	Chat GPT	Gemini	Llama	p value
Modified DISCERN, median (IQR)	3 (0)	3 (2)	4 (1)	<0.001^b^
EQIP, mean ± s.d.	68.7 ± 2.1	54.2 ± 1.3	73.8 ± 2.2	<0.001^a^
FGKL, mean ± s.d.	23.3 ± 1.8	24.5 ± 2.0	23.7 ± 2.6	0.167^a^
FRES, mean ± s.d.	37.7 ± 7.9	35.4 ± 9.6	34.2 ± 8.2	0.366^a^

*s.d*. standard deviation, *IQR* interquartile range, *EQIP* the ensuring quality information for patients, *FGKL* Flesch–Kincaid grade level, *FRES* Flesch reading ease score.

^a^One-way anova.

^b^Kruskal-Wallis.

The chatbot responses were further categorized into three subgroups —general information, surgical treatment and myths/misconceptions— and analyzed accordingly. In all three categories, the Llama chatbot demonstrated statistically significant higher EQIP and Modified DISCERN scores compared to the other chatbots (p < 0.001) (Table 3).

**Table 3:** Subgroup analysis of the scoring system between the chatbots^a^.

	EQIP score	p value	Modified DISCERN	p value	FKGL	p value	FRES	p value
General Inf.		**<0.001**^b^		**<0.001**^b^		0.694		0.267^b^
ChatGPT	68.4 ± 1.1		3(1)		23.4 ± 1.6		41.1 ± 9.2	
Gemini	55.2 ± 1.4		1(2)		24.1 ± 2.9		33.8 ± 8.2	
Llama	71.1 ± 1.6		4(0)		23.2 ± 1.9		35.3 ± 9.8	
Treatment		**<0.001**^b^		**0.003**^b^		0.106		0.150^b^
ChatGPT	65.7 ± 2.2		3 (1)		22.2 ± 2.2		40.0 ± 7.5	
Gemini	55.2 ± 1.6		1 (2)		24.7 ± 2.1		33.4 ± 10.6	
Llama	73.6 ± 4.1		4(1)		25.1 ± 1.8		32.9 ± 10.5	
Myths		**<0.001**^b^		**0.004**^b^		0.288		0.340^b^
ChatGPT	71.1 ± 1.7		3(0)		24.1 ± 1.2		33.3 ± 5.5	
Gemini	52.6 ± 2.5		3(0)		24.6 ± 1.2		38.1 ± 10.3	
Llama	76.3 ± 2.1		3(1)		23.1 ± 3.3		34.4 ± 5.2	

*EQIP* The Ensuring Quality Information for Patients, *FGKL* Flesch–Kincaid Grade Level, *FRES* Flesch Reading Ease Score.

^a^Data are presented mean ± standard deviation or median (interquartile range).

^b^Kruskal-Wallis test.

Bold values indicate statistically significant results.

## Discussion

This study represents the first comprehensive evaluation of chatbot-generated responses specifically within the context of penile enhancement, thereby contributing significantly to the rapidly growing field of AI-driven health information. Comparative analysis of the three chatbot models revealed marked disparities in response quality, with Llama demonstrating superior performance in content quality compared to both ChatGPT and Gemini, further highlighting its potential for providing more reliable and informative responses. Specifically, Llama achieved significantly higher scores on both the Modified DISCERN and EQIP scoring methods and performed even better when the subgroups of questions related to penis enhancement (general information, surgical treatment and myths) were compared, demonstrating its ability to consistently deliver more accurate and higher quality content. These findings underscore Llama’s remarkable capacity to consistently deliver more detailed, accurate, and reliable information, positioning it as a more robust and trustworthy resource for addressing complex and sensitive inquiries related to penile enhancement, particularly in cases where precise, high-quality content is crucial for informed decision-making and patient guidance.

A study reported that the responses generated by ChatGPT, Perplexity, Chat Sonic and Microsoft Bing AI on urological cancers lacked actionable guidance for users, raising concerns about their practical applicability [16]. Another study evaluating ChatGPT’s accuracy on urological guideline-based questions found that only 60% of responses were appropriate, with 25% showing inconsistencies [15]. This poor performance was reflected by low Brief DISCERN scores (mean 16.8 ± 3.59; 54% met the quality threshold), largely due to ChatGPT’s failure to provide or accurately cite sources (92.3% error rate). This raises concerns about its reliability as a urology information resource [15]. Another study compared responses to 25 andrology cases from 32 experts, 18 residents, and 3 chatbots (ChatGPT v3.5, v4, and Bard) using a Likert scale (0 = incorrect/no response, 1 = partially correct, 2 = correct) [23]. Analysis of mean scores revealed that experts achieved the highest performance (11), followed by ChatGPT v4 (10.7, p = 0.6475), residents (9.4), ChatGPT v3.5 (9.5, p = 0.0062) and Bard (7.2, p < 0.0001). A statistically significant difference in performance was observed between residents and Bard (p = 0.0053) [23]. These performance disparities between chatbot models and healthcare professionals raise concerns regarding chatbot reliability for clinical application [23]. The modified DISCERN assessment in our study yielded median scores of 3 (IQR 0) for ChatGPT, 3 (IQR 2) for Gemini and 4 (IQR 1) for Llama. This difference in scores suggests that Llama’s ability to cite sources is a contributing factor to its higher performance.

Various scoring systems, such as the Patient Education Material Assessment Tool (PEMAT), global quality score (GQS), Likert Scales and EQIP have been applied to evaluate the quality of health information provided by AI-based chatbots [13, 24–26]. EQIP is a scoring system used by healthcare professionals to evaluate written health information, demonstrating established validity, reliability and utility [20]. It has been frequently employed in recent articles evaluating AI-based chatbots [12, 13, 27]. In a study examining ChatGPT’s responses regarding hepatobiliary diseases, EQIP scores were calculated for the entirety of the texts as well as for three subsections: content, identification and structure. The median score for all 36 items was 16 (IQR 14.5–18), while when divided into subsections, the median scores were observed to be 10 (IQR 9.5–12.5), 1 (IQR 1–1) and 4 (IQR 4–5), respectively [27]. It has been reported that, in this study, the comparison of the breakdown of the scores achieved reveals that ChatGPT scores higher in the content domain but lower in the identification and structure domains [27]. In studies where ChatGPT’s responses for erectile dysfunction and premature ejaculation were scored using the EQIP tool, the average scores of the texts were determined to be 40.0 ± 4.2 and 45.93 ± 4.34, respectively, indicating that while the quality of the information provided varied, overall the scores were relatively low [12, 13]. These findings highlight the need for further evaluation of AI-generated content to ensure its accuracy and reliability in medical contexts. [12, 13]. In our study, the mean EQIP scores for ChatGPT, Gemini, and Llama were reported as 68.7 ± 2.1, 54.2 ± 1.3, and 73.8 ± 2.2, respectively. Particularly, we believe that the higher EQIP score for ChatGPT compared to other studies in the literature may be attributed to differences in the evaluation criteria, the complexity of the medical topics addressed or advancements in the model’s training and fine-tuning processes over time.

In online health information texts, readability and understandability are crucial for individual health as this ensures that patients are properly guided [28]. Despite their remarkable capabilities, it has been reported that chatbots have significant limitations in terms of readability and understandability when used as medical information sources, and that improvements should be made before they are adopted for this use [17]. The FKGL is a readability test that indicates the U.S. school grade level required to understand a text, with higher scores suggesting more complex language [19]. The FRES measures the ease of reading a text, where higher scores represent easier readability [19]. These tests are commonly used to assess the accessibility of written content for different audiences [19]. An analysis of ChatGPT’s responses on penile prosthesis implantation revealed FKGL scores ranging from 14.04 to 17.41 and FRES between 9.8 and 28.39, indicating a readability level suitable for college audiences [29]. In a study examining responses from five different chatbots (ChatGPT, Bard, Bing, Ernie, Copilot) on erectile dysfunction, it was found that ChatGPT had the statistically highest mean FKGL score (14.3 ± 1.7), making its understandability the most difficult (p < 0.001) [13]. In terms of readability, Bard emerged positively with the highest mean FRES (53.9 ± 21.5 p < 0.001); however, its understandability was reported to be difficult, though not as much as ChatGPT [13]. These findings highlight the need to improve chatbot output to ensure it is both understandable and user-friendly, particularly in medical contexts. In our study, no statistically significant difference was found between the mean FKGL scores and FRES for ChatGPT, Gemini and Llama. Due to FKGL scores being above 16, it was determined that the texts are aimed at a “college graduate/academic level” audience. When interpreting the FRES, it was revealed that the texts correspond to stage 4, indicating “difficult readability”.

### Limitations

One of the main limitations of this study is that the evaluation tools used were not specifically designed for AI chatbot assessments. This highlights the need for new, specific scoring systems to accurately and comprehensively evaluate AI-based chatbots’ health information. Moreover, AI chatbots were not compared with established Patient Education Materials (PEMs). This comparison could have provided a more comprehensive assessment of their effectiveness in delivering health-related information.

Additionally, the limited number of chatbot models examined in our study and their varying performance findings suggest that these may not reflect the quality of all chatbot models. Furthermore, the fact that only English responses were evaluated means that potential quality differences for chatbots in different languages was not captured.

## Conclusion

This study highlights the performance differences among various AI chatbot models in delivering health-related information, particularly in the field of penis enhancement. Although Llama emerged as the most reliable and informative source, it should be considered that the decision was made based on qualitative criteria. Therefore, a comparative analysis could be conducted using validated multiple-choice medical questions with only one correct answer per question, developed by an expert panel, to provide objective and absolute performance metrics.

Additionally, it was found that the readability and understandability of AI chatbots is quite challenging. Future research should focus on enhancing the reliability of chatbot responses in medical fields, making them more understandable and accessible to a broader audience and the development of a universally applicable evaluation tool for AI chatbot responses.

## Data Availability

The existing data used in this study are available for sharing upon request.
